# Supplementary material for: Cortical and medullary oxygenation evaluation of kidneys with renal artery stenosis by BOLD-MRI
Source: PLoS One. 2022 Mar 10;17(3):e0264630. doi: 10.1371/journal.pone.0264630 (PMC8912187; doi:10.1371/journal.pone.0264630)
Supplement: Attachment — Submitted filename: Response to Reviewers.docx [file pone.0264630.s001.docx]

I’m very grateful for the reviewers' affirmation and further suggestions of our research. The reviewer has mentioned a recent consensus based technical recommendations published in Nov. 2019, named as Consensus‑based technical recommendations for clinical translation of renal BOLD MRI [PMID: 31768797]. We conducted our research from 2017 to 2018, and finished our paper in 2019. We did not refer to the recommendations in our experiment. But I read that review carefully after the reviewer recommend it to me. Fortunately, we keep pace with these top researchers. After a comprehensive survey, they presented final recommendations on renal BOLD MRI data acquisition, analysis and interpretation including 21 items. I check up on our research and find that we reach consensus on at least 17 out of 21 items (81%). The rest of items including other “red light” topics with a 50/50% split in researchers will be useful for us to draw perspectives and design further experiments. Here are the answers to each Specific point raised by the reviewers below：

1. There are several methods of ROI selection in BOLD MRI: the small manual ROI, the large manual ROI, the hybrid ROI and the whole parenchyma analysis method (the compartmental method). Firstly, contrast-enhanced CT images, as used in the hybrid method, are usually unavailable in human research, while their coregistration to MRI is time consuming and requires skill. Secondly, the large ROI method is more vulnerable than the small ROI to partial volume effect of blurry cortico-medullary border. Finally, earlier investigations revealed that the mean R2* values obtained by different ROI methods are close [PMID: 22183077]. The whole parenchyma analysis method has an advantage in comparative studies of pre- to post-drug, because of less time consuming than the manual method, especially in coregistration. But in oxygenation evaluation of kidneys with renal artery stenosis (RAS), we didn’t need to observe the response of R2* value to any drug or intervention. On the contrary, the whole parenchyma analysis method may be sensitive to abdominal susceptibility artifact (ASA), while the manual ROI method could remove it obviously. There were some other reasons made us abandoned the whole parenchyma analysis method, such as its statistical nature. When the amount of available data was limited, the histogram curve was not smooth enough, which led the accuracy might be compromised.

2. The ROIs were drawn on the original BOLD-MRI image, which showed a clear anatomical structure. Then, the ROIs were projected automatically to the T2* mapping image to obtain the T2* value (milliseconds) of the ROI. Then we got R2* values (the reciprocal of T2* values) by program 2. So all the values we shown in results of the paper are R2* values, including mean values, max and min values in Figure 1, just as the consensus‑based technical recommendations recommended. T2* values were just presented in procedure.

3. Each patient underwent renal artery ultrasound examination in the research. Some of them underwent computed tomography angiography (CTA) or magnetic resonance angiography (MRA) as well. But others could only afford the ultrasound examination because of avoiding of radiographic contrast nephropathy. We have tried our best to make the decision of RAS degree accuracy via combined different methods of renal artery examination. Besides, we made a discussion about different methods of renal artery examination in our earlier paper, Comparative study of the diagnostic value of non-enhanced magnetic resonance angiography and ultrasound in the degree of renal artery stenosis in patients with renal insufficiency (Doi:CNKI:SUN:XFXZ.0.2021-03-012.) .

4. The mechanism of R2* values variation of diabetics is not clear. We are planning to study it specifically in next research. This is the reason we excluded diabetics in this research.

5. I followed reviewer’s advice and deleted the redundant description.

6. We did acquired 4 slices, but only used only one slice data for each patient. Just as reviewer mentioned in question 1, adequate cortico-medullary differentiation is necessary for ROI selection. But some oblique coronal slices which far from the renal hilus could not obtain adequate cortico-medullary differentiation leading to volume averaging. So we selected a slice with a most clear demarcation of the renal cortex and medulla out of 4 slices to drawn ROIs. Consequently, we got less data as well as more accurate data and more sensitive results. Benefitting of reviewer’s advice, we have noticed that more slices are recommended [PMID: 22183077]. We would consider this point in the further research.

7. We use two custom Matlab codes in post-scan processing for different aims. Get the TE slice (3.38 ms) by code 1. Draw ROIs and obtain R2* values by code 2. Corresponding simplifications have been completed in manuscript. We used Image J in the preliminary study. The usage of TLCO method and histogram analysis has been discussed in answer 1.

8. Comparison of regional differences shown some positive results in patients. It does be quite possible that they also have other disease leading to more regional differences. We are also curious that how does variation of regional R2* values occurred in RAS patient. We are considering to do further study in these patients or distal renal artery stenosis patients.

9. Corresponding modifications have been completed in manuscript.

10. Corresponding modifications have been completed in manuscript.

11. Renal artery stenosis may be not considered as peripheral artery disease. Corresponding modifications have been completed in manuscript.
