# Supplementary material for: Cortical and medullary oxygenation evaluation of kidneys with renal artery stenosis by BOLD-MRI
Source: PLoS One. 2022 Mar 10;17(3):e0264630. doi: 10.1371/journal.pone.0264630 (PMC8912187; doi:10.1371/journal.pone.0264630)
Supplement: Attachment — Submitted filename: Response to Reviewers.docx [file pone.0264630.s002.docx]

There are still several issues which need to be settled after the prior version. Here are the answers to each specific point raised by the reviewers below：

1. A combined image showing all the ROIs of the anatomical image and the T2* map is very helpful, which could improve the legibility and reliability of the figure. So we have modified “Figure 1 and 2” into “Figure 1” with the anatomical image, the T2* map and all the ROIs. Absolutely, we have done the same with “Figure 3” by including the anatomical image and showing all the ROIs, name as “Figure 2”, just as the reviewer instructed. A T2* map with a gray scale bar is also an inspirational idea, but the R2* values of many organizations in the whole FOV will be extremely out of range, leading to an extremely large scale, and the intuitive readability of the scale bar will be poor. Here is an illustration below (unit: ms).

2. In the “Figure 1” of prior version, “mean value shown is for R2* while Max and Min values are T2*” do exist, we have update new figures with all the ROIs including anatomical image and T2* map.

3. Changes in renal parenchymal oxygenation caused by renal artery stenosis are sometimes only local. At this time, if the T2* values are measured and averaged by the whole kidney, the changes of T2* values in local renal tissue may be covered up. On the contrary, it is possible to miss lesions when measuring T2* value with multiple small ROIs, so we try to value the upper, middle and lower parts of the kidney separately to avoid missing lesions. The two measurements require a balanced strategy. The choice of reporting only one slice out of four acquired slices might miss lesion which located in other slices, but differences between groups have already discovered by middle slices. Meanwhile, the cortical-medulla boundary of the kidney is clearer in the middle layer, and at the same time, the partial volume effect of the marginal layer is avoided, resulting in inaccurate T2* value.

4. We remove “Figure 4” because of medulla R2* values shown in “Figure 4” were already included in Table 5, just as the reviewer mentioned.
